# Blocking the A_2B_ adenosine receptor alleviates myocardial damage by inhibiting spleen-derived MDSC mobilisation after acute myocardial infarction

**DOI:** 10.1080/07853890.2022.2084153

**Published:** 2022-06-08

**Authors:** Zongying Yu, Yang Ling, Qiancheng Xu, Yuhan Cao, Shengxing Tang, Cong Fu

**Affiliations:** a Department of Cardiology, Yi Ji Shan Hospital Affiliated to Wan Nan Medical College, Anhui, China; b Anesthesia Laboratory and Training Center of Wan Nan Medical College, Anhui, China; c Department of Critical Care Medicine, Yi Ji Shan Hospital Affiliated to Wan Nan Medical College, Anhui, China; d Department of Nephrology, Yi Ji Shan Hospital Affiliated to Wan Nan Medical College, Anhui, China; e Key Laboratory of Non-coding RNA Transformation Research of Anhui Higher Education Institution (Wan Nan Medical College), Anhui, China

**Keywords:** Acute myocardial infarction, MDSCs, A_2B_ adenosine receptor, spleen

## Abstract

**Background:**

Myeloid-derived suppressor cell (MDSC) mobilisation is an important immune event in acute myocardial infarction (AMI). The A_2B_ adenosine receptor (A_2B_AR) plays key role in regulating MDSC function, but its specific involvement in MDSC mobilisation in AMI remains unclear.

**Methods:**

In AMI patients, the circulating MDSC ratio and A_2B_AR mRNA expression were measured. A mouse AMI model was established by left anterior descending coronary artery (LADCA) ligation. MDSCs were analysed by FACS and immunofluorescence staining (of heart tissue). A_2B_AR mRNA expression was assessed by qRT-PCR. Myocardial injury was detected by HE staining. Myocardial cell apoptosis was analysed by immunohistochemistry. Cardiac systolic function was evaluated by transthoracic echocardiography.

**Results:**

In AMI patients, the circulating MDSC ratio was increased and positively correlated with A_2B_AR mRNA expression (*r* = 0.86, *p* < 0.01). In AMI model mice, the percentage of MDSCs was increased in the circulation and infarcted heart and decreased in the spleen. MRS-1754-mediated A_2B_AR inhibition decreased the MDSC ratio in the circulation and infarcted heart and prevented the decrease in MDSC number in the spleens of mice with AMI. A_2B_AR blockade inhibited myocardial cell apoptosis, alleviated myocardial inflammatory injury, and improved myocardial systolic function in the AMI mouse model. Similar results were found in mice after splenectomy. Additionally, spleen-derived MDSC injection increased the MDSC ratio in the infarcted heart, increased myocardial cell apoptosis, aggravated myocardial injury, and decreased cardiac systolic function in mice with AMI.

**Conclusion:**

Blocking A_2B_AR alleviates myocardial damage and improves myocardial systolic function through inhibition of spleen-derived MDSC mobilisation after AMI.

**Key Messages**
- Spleen-derived MDSC mobilisation aggravates myocardial inflammatory injury within 24 h of AMI.
- A_2B_AR promotes spleen-derived MDSC mobilisation within 24 h of AMI.
- Blocking A_2B_AR improves myocardial systolic function through inhibition of spleen-derived MDSC mobilisation.

## 1. Introduction

Acute myocardial infarction (AMI) is a cardiac emergency involving myocardial necrosis caused by unstable coronary artery ischaemic syndrome, and remains a leading cause of death and disability worldwide [1–3]. Recent studies on AMI have shown that a persistent proinflammatory reaction plays a vital role in determining the myocardial infarction (MI) size and subsequent left ventricle remodelling [4–6]. Undoubtedly, regulating the inflammatory response to AMI is a potential therapeutic strategy for limiting the MI size and improving outcomes following AMI [5].

Myeloid-derived suppressor cells (MDSCs) are a heterogeneous population of myeloid cell progenitors and precursors and are generated during biological stress, including tissue damage and inflammation [7–9]. Mouse MDSCs are CD11b^+^ and Gr-1^+^, while human MDSCs are mostly CD11b^+^ and CD14^-^ [9–11]. These cells were first described more than 20 years ago in patients with cancer [12], and increasing evidence indicates that MDSCs are universal regulators of immune function in various heart diseases, such as aging-related cardiac fibrosis [13], acute coxsackievirus B3-induced myocarditis [14], and sepsis-induced myocardial injury [15]. A recent study suggested that enhanced infiltration of MDSCs by advanced glycation end-products contributes to aggravation of myocardial injury in mice with AMI [16]. Our previous study found that MDSCs participate in all pathogenic processes of acute coronary syndrome by gradually differentiating into foam-like and neutrophil-like cells [17]. Therefore, improving our understanding of MDSC mobilisation may provide clues for improving therapeutic strategies for AMI.

A_2B_ adenosine receptor (A_2B_AR), a member of the adenosine receptor family, plays critical roles in tumours and cardiovascular diseases through its proangiogenic effects [18–21]. Blockade of A_2B_AR attenuates cardiac remodelling, ameliorates left ventricular dysfunction, and improves cardiac outcomes after AMI in animal models [22–24]. In many tumour models, A_2B_AR blockade or knockout reduces tumour-associated MDSC accumulation and leads to a significant delay in tumour growth [25–29]. However, whether A_2B_AR can regulate MDSC mobilisation in AMI remains unclear.

Therefore, this study was designed to confirm whether MDSCs infiltrate myocardial tissue and further lead to myocardial injury during AMI. Furthermore, we aimed to investigate the origin of MDSCs. Moreover, a preliminary mechanism of and possible strategies for intervening in MDSC mobilisation are discussed.

## 2. Methods

### 2.1. AMI patients

Blood samples were collected from the peripheral veins of 10 AMI patients who underwent emergency coronary angiography (CAG) and were hospitalised in the cardiology ward of Yi Ji Shan Hospital affiliated to Wan Nan Medical College. Ten age- and sex-matched patients without coronary artery disease, as confirmed by CAG, were included as controls. There was no significant difference in the basic data between the 2 groups (Supplementary Table 1). All participants agreed to participate in the study and provided written informed consent. The study protocol was approved by the Ethics Committee of Yi Ji Shan Hospital.

### 2.2. Mice grouping and AMI model

Male C57BL6 mice (age: 6–8 weeks, weight: 20–25 g) were used according to the guidelines of the Animal Ethics Committee of Yi Ji Shan Hospital affiliated to Wan Nan Medical College. Mice were randomly divided into 6 groups. Control group (*n* = 5): mice did not receive any operations. Sham group (*n* = 5): mice underwent control operation without coronary artery ligation. AMI group (*n* = 10): mice underwent LADCA ligation. A_2B_AR blocking group (*n* = 10): mice received A_2B_AR antagonist MRS1784 (1 mg/kg) [30] intraperitoneally injection before LAD ligation. Splenectomy group (*n* = 10): mice underwent splenectomy before LAD ligation. MDSCs injection group (*n* = 10): the splenectomy mice immediately received isolated MDSCs (2 × 10^5/^ml) injection *via* the medial canthus vein before LADCA ligated.

AMI was induced by ligation of the left anterior descending artery (LADCA) [31]. Briefly, the mice were anaesthetised by intraperitoneal injection of sodium pentobarbital (35 mg/kg) (Sigma-Aldrich, USA) and intubated. Then, the LADCA was ligated proximally with 7-0 silk sutures via a left thoracotomy incision. Paleness of the anterior wall of the left ventricle indicated successful induction of AMI. All mice were housed in an SPF animal facility with unrestricted access to food and water.

Splenectomy was performed as previous described [32]. A skin incision (approximately 0.5 cm) is made in vertical midline abdomen of adequate anaesthetised mice. After ligation of the relevant vessels, spleen was removed to separate from the stomach. Then, the midline fascial defect and skin were closed with interrupted 4-0 Vicryl suture with using Wire Twister for Neurosurgery.

### 2.3. Cell preparation

Mononuclear cells were isolated from the peripheral venous blood of patients on hydroxypropyl methylcellulose by centrifugation at 500×*g* for 20 min. White blood cells were isolated from the peripheral blood of mice by using erythrocyte lysate (BD Biosciences, USA). Mononuclear cells were obtained from the mouse spleen and heart with a gentleMACS Dissociator.

MDSCs were also isolated from the mouse spleen by using a mouse MDSC isolation kit (Miltenyi Biotec, Germany) according to the manufacturer's instructions. Disrupt spleen in PBS containing 2% foetal bovine serum (FBS). Then, the cell suspension was filtered through a 70 μm mesh nylon strainer to removed aggregates and debris. After centrifugation (300 × g for 10 min), the cells were resuspended at 1 × 10^8^ nucleated cells/mL in medium. Add FcR blocker, isolation cocktai and RapidSpheres™ to the resuspended cells suspension in turn, mix and incubate for 5 min at room temperature. Subsequently, MDSCs were isolated through a magnet. FACS was used to evaluate the separation efficiency of MDSCs isolated from the spleen. We found that the isolation efficiency was approximately 59.4% (Supplementary Figure 1). At last, the isolated MDSCs were resuspend and cultured at 2 × 10^5^ nucleated cells/mL in recommended medium.

**Figure 1.**
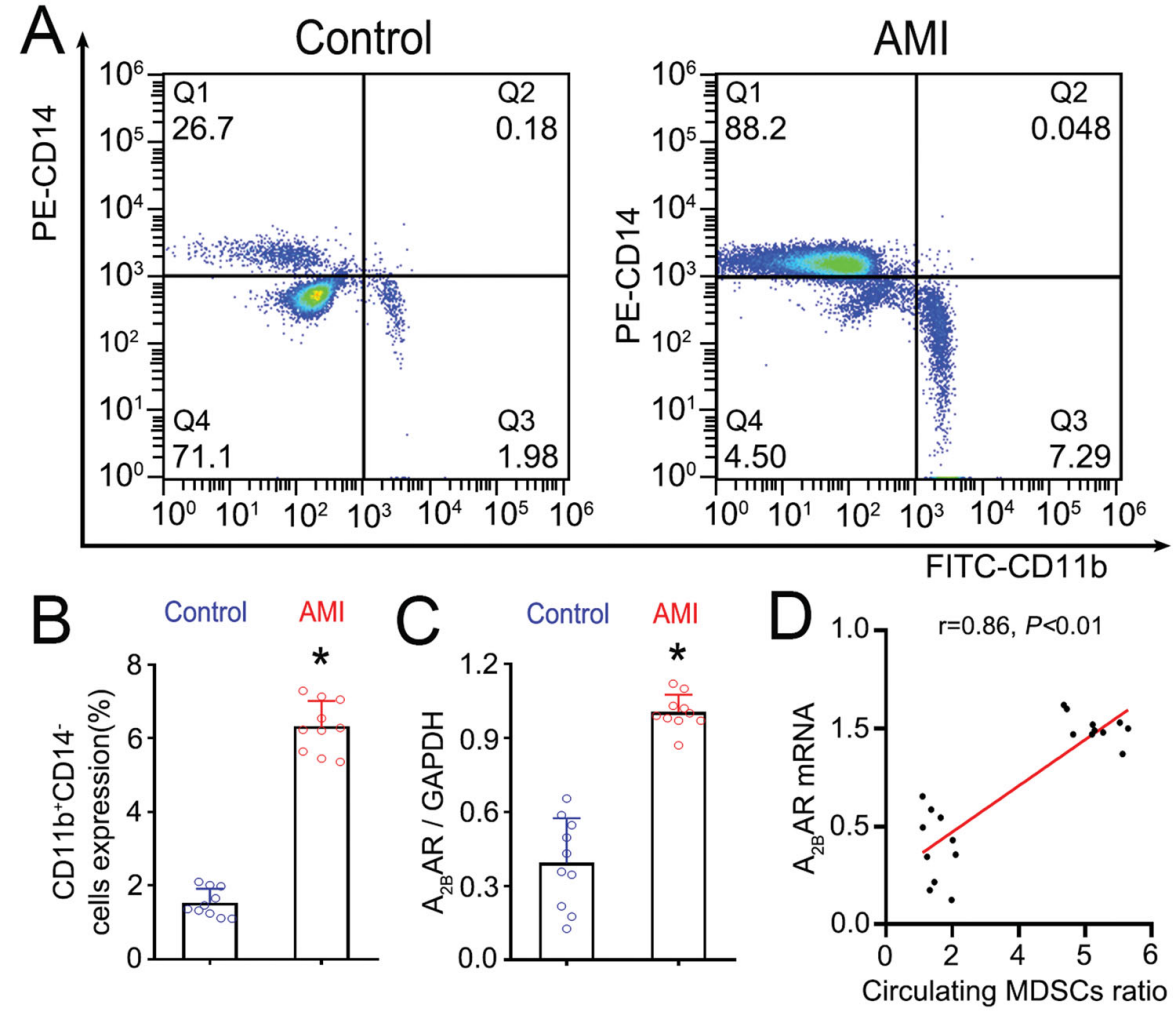
The circulating MDSC ratio is correlated with A_2B_AR mRNA expression in AMI patients. (A) Representative flow cytometry images of MDSCs in the circulation of patients. (B) Mean fluorescence intensity of CD11b^+^ CD14^–^ cells. (C) Expression of A_2B_AR mRNA in the PBMCs of AMI patients. (D) Correlation of the circulating MDSC ratio and A_2B_AR mRNA expression in the mononuclear cells of AMI patients. For each group: *n* = 10, three independent experiments. Statistical analysis was performed using Student’s *t* test (B, C) or Pearson’s analysis (D). *p < 0.001 vs. the control group.

### 2.4. FACS analysis

Isolated cells were fixed in 1% paraformaldehyde (Sigma-Aldrich, USA) and incubated with PE-conjugated antihuman CD14, FITC-conjugated antihuman CD11b, PE-conjugated antimouse CD11b, and FITC-conjugated antimouse Gr-1 antibodies for 1 h on ice. After washing three times with 1% BSA in PBS, the cells were analysed with a FACScan flow cytometer to detect human MDSCs (CD11b^+^ CD14^–^) and mouse MDSCs (CD11b^+^ Gr-1^+^). All antibodies and the FACScan flow cytometer were purchased from BD Biosciences.

### 2.5. Immunofluorescence staining

Twenty-four hours later, the mice were sacrificed by intraperitoneal injection of sodium pentobarbital (130 mg/kg). Heart tissue specimens were harvested and prepared for immunofluorescence staining [15]. Heart tissue was fixed in 10% formaldehyde (Sigma-Aldrich, USA) and then embedded in paraffin. The tissue sections (2 μm thick) were incubated with PE-conjugated antimouse CD11b and FITC-conjugated antimouse Gr-1 antibodies and then with DAPI (1 μm/ml) (Roche) in the dark for 15 min. Finally, mouse MDSCs (CD11b^+^ Gr-1^+^) were detected by inverted phase contrast fluorescence microscopy (Olympus, Japan).

### 2.6. A2bar mRNA expression

Total RNA was isolated from mononuclear cells using the TRIzol method according to the manufacturer’s protocol. The concentration and purity of RNA were assessed by determining the relative absorbance ratio at 260/280 with a NanoDrop 2000 spectrophotometer (Thermo Scientific, USA). cDNA was synthesised using a PrimeScript^TM^ RT reagent kit (TAKARA, Japan) with gDNA Eraser. A_2B_AR (sense: 5′-CTCCATCTTCAGCCTTCT-3′; anti-sense: 5′-ACCAAACTTTTATACCTGAGC-3′) and GAPDH (sense: 5′-GGTGAAGGTCGGAGTCAACGGATTTGGTCG-3′; antisense: 5′-GGATCTCGCTCCTGGAAGATGGTGATGGG-3′) primers were used. A_2B_AR expression was normalised to GAPDH expression and calculated by the 2^−ΔΔCt^ method.

### 2.7. Histopathology and immunohistochemical staining

Hematoxylin–eosin (HE) staining was performed to evaluate heart tissue damage. Paraffin heart tissue sections were stained with HE by using a HE staining kit (Beyotime, China) according to the manufacturer’s protocol. Morphological changes in myocardial tissue were assessed using an optical microscope. Cardiac muscle structure, myocardial cell fibrils swelling and arrangement, and granulocytes infiltration in heart specimens were observed and measured [33]. A semiquantitative score was obtained to assess the degree of myocardial injury.

Tetrazolium chloride (TTC) staining was performed to detect the area of myocardial infarction [34]. Following MI, myocardial tissues were rapidly removed and sliced into 2 mm sections. Then, the sections were incubated TTC solution (Sigma, USA) at 37 °C for 30 min. Infarct areas (white) in the myocardial tissues were photographed and measured by Image J software. The ratio of the infarct area to the corresponding cardiac cross-sectional area was calculated and compared the differences among groups.

Immunohistochemical staining was performed to detect the nuclei of apoptotic myocardial cells by using a TUNEL staining kit (Beyotime, China) according to the manufacturer’s protocol. The number of apoptotic cells with TUNEL-positive nuclei was counted.

### 2.8. Echocardiography

Twenty-four hours after MI model establishment, the cardiac systolic function of the mice was evaluated by transthoracic echocardiography. The ejection fraction (EF), left ventricular shortening (LVFS), left ventricular end diastolic diameter (LVIDd), and left ventricular systolic inner diameter (LVIDs) were calculated.

### 2.9. Statistical analysis

Statistical analyses were performed with GraphPad Prism version 9.0. All continuous variables are expressed as the mean ± SD. Student’s *t* test (2 groups) or one-way ANOVA (≥3 groups) was used to assess differences among groups. Pearson correlation analysis was used to assess the correlation between the circulating MDSC ratio and A_2B_AR mRNA expression. *p* < 0.05 was considered statistically significant.

## 3. Results

### 3.1. The circulating MDSC ratio is correlated with A_2B_AR mRNA expression in AMI patients

The circulating MDSC ratio was significantly increased in AMI patients compared to controls (Figure 1(A,B)). We further assessed A_2B_AR mRNA expression in mononuclear cells by qRT-PCR. The results showed that A_2B_AR mRNA expression was also increased in AMI patients compared to controls (Figure 1(C)). Pearson correlation analysis showed that the circulating MDSC ratio was positively correlated with A_2B_AR mRNA expression (*r* = 0.86, *p* < 0.001; Figure 1(D)).

### 3.2. A_2B_AR regulated MDSCs mobilisation after AMI

**Figure 2.**
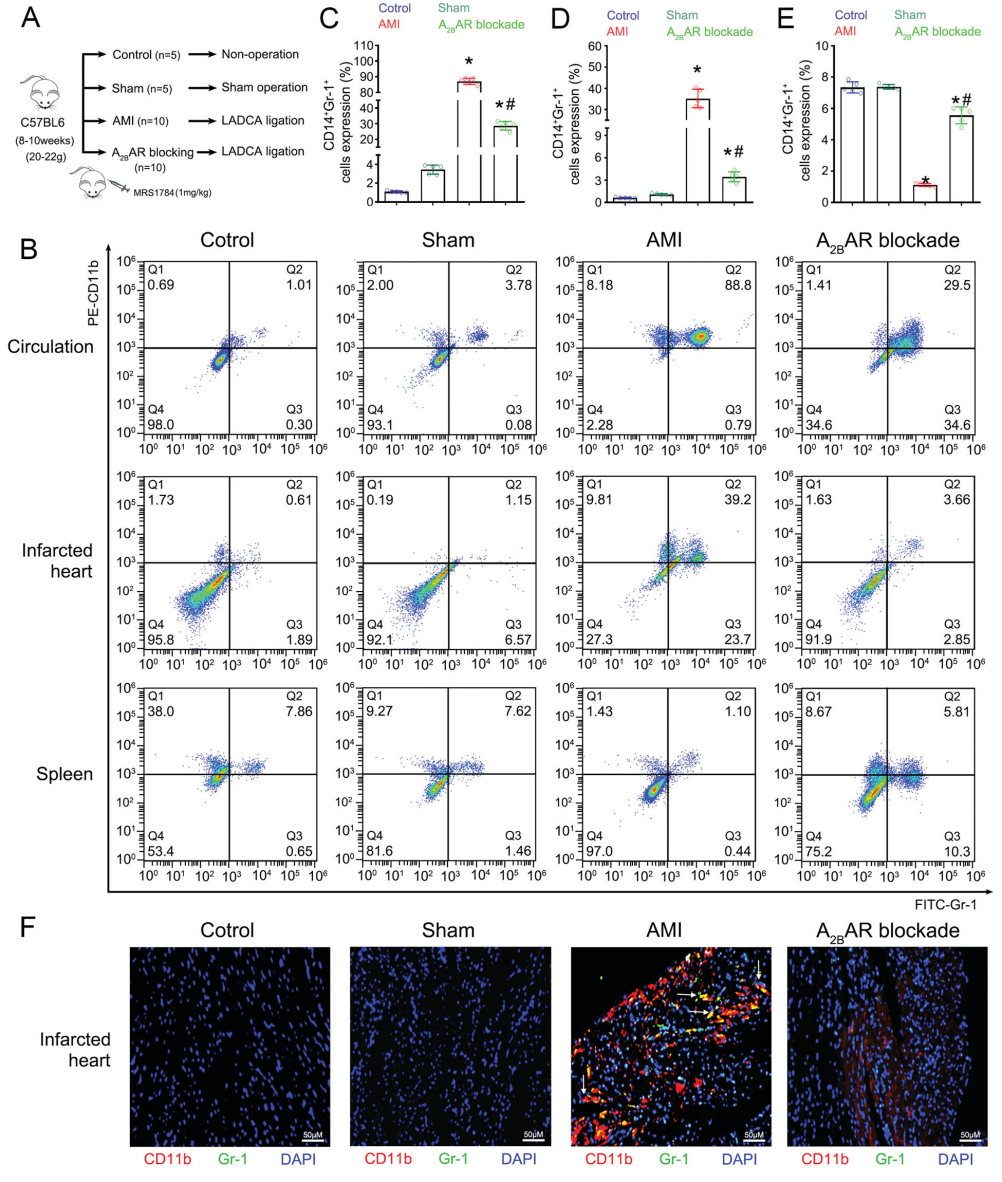
A_2B_AR regulated the mobilisation of MDSCs after AMI. (A) The different groups of mice. Male C57BL6 mice (age: 8–10 weeks, weight: 20–22 g) were randomly divided into 4 groups. The control group (*n* = 5) did not undergo any operation. The sham group (*n* = 5) underwent sham operation without coronary artery ligation. The AMI group (*n* = 10) underwent LADCA ligation. The A_2B_AR blockade group (*n* = 10) received intraperitoneal injection of the A_2B_AR antagonist MRS1784 (1 mg/kg) before LADCA ligation. (B) Representative flow cytometry images of MDSCs in the circulation, infarcted heart, and spleen among the 4 groups. (C) Mean fluorescence intensity of CD11b^+^ CD14^–^ cells in the circulation of mice. (D) Mean fluorescence intensity of CD11b cells in the infarcted mouse heart. (E) Mean fluorescence intensity of CD11b^+^ CD14^–^cells in the mouse spleen. (F) Representative immunofluorescence staining images of CD11b (red), Gr-1 (green), and DAPI (blue) staining in the infarcted mouse heart and merged images (right column). For each group: *n* = 5 (A–E), *n* = 3 (F); three independent experiments. Statistical analysis was performed using one-way ANOVA. *p < 0.001 vs. the control and sham groups; *p < 0.001 vs. the AMI group.

To explore whether A_2B_AR regulates the mobilisation of MDSCs after AMI, the A_2B_AR antagonist MRS1784 (1 mg/kg) (Selleck, USA) was injected intraperitoneally into mice before LADCA ligation (Figure 2(A)). We found that 24 h after LADCA ligation, the MDSCs ratios in the circulation (Figure 2(B,C)) and infarcted heart (Figure 2(B,D)) were significantly increased in the AMI group compared to the control and sham group.

However, the ratio of MDSCs in the spleen was significantly decreased in the group that underwent LADCA ligation compared to the control and sham groups (Figure 2(B,E)). A_2B_AR blockade significantly decreased the MDSC ratio in the circulation (Figure 2(B,C)) and in infarcted heart (Figure 2(B,D)) after LADCA ligation. However, the decrease in the MDSC ratio in the mouse spleen was significantly prevented by A_2B_AR blockade (Figure 2(B,D)).

Immunofluorescence revealed that the MDSC ratio was increased in the infarcted mouse heart and that this increase was abolished by A_2B_AR blockade (Figure 2(F), Supplementary Figure 2). All of the data suggested that A_2B_AR blockade effectively prevents the reduction in the MDSC ratio in the spleen and decreases the MDSC ratio in the circulation and infarcted heart.

### 3.3. A_2B_AR blockade alleviated myocardial injury after AMI

**Figure 3.**
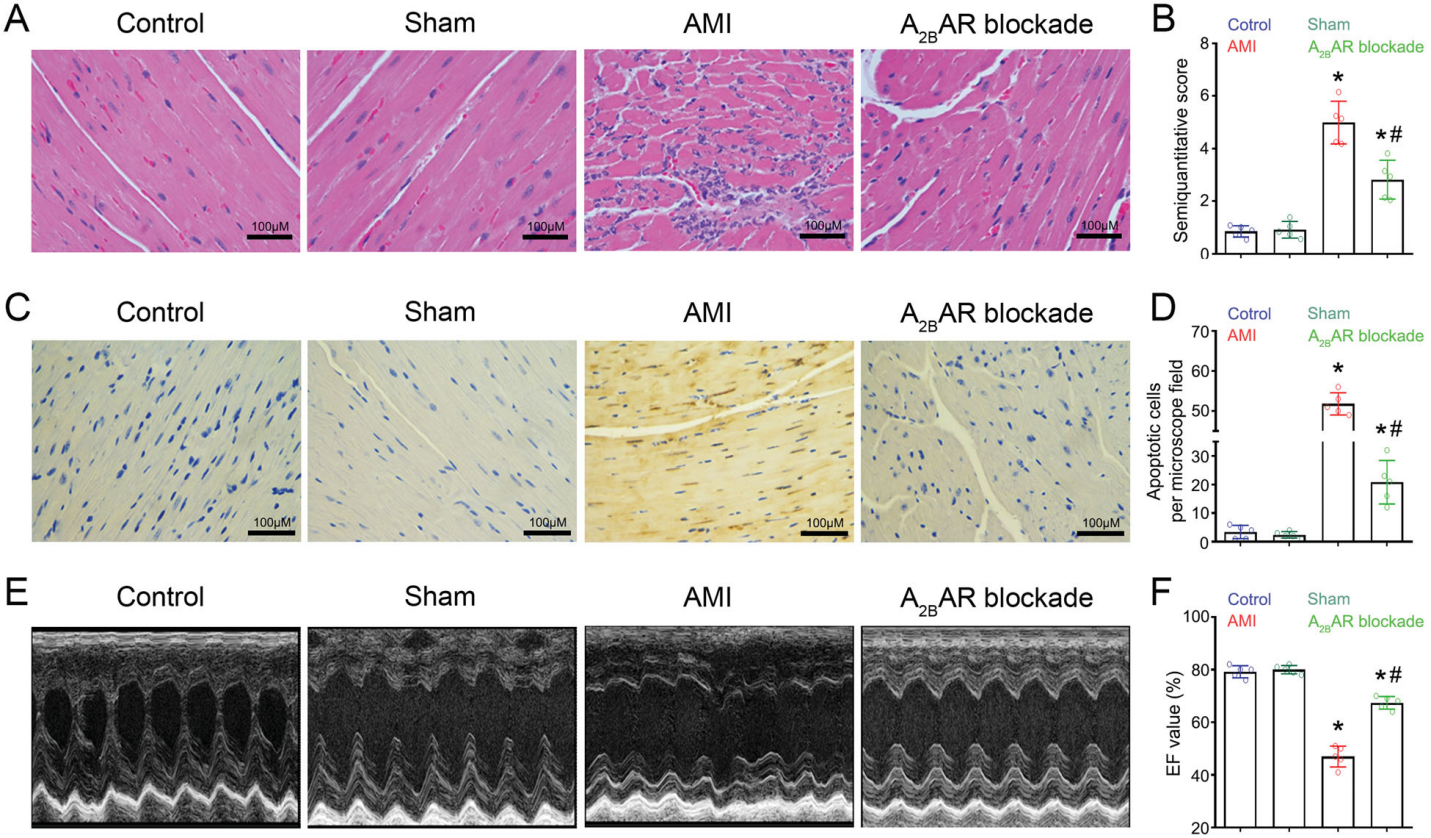
A_2B_AR-mediated MDSC mobilisation led to myocardial injury after AMI. (A) Representative HE staining images of the infarcted mouse heart. (B) Mean semiquantitative score of the mouse myocardium. (C) Representative images of myocardial apoptosis in the infarcted mouse heart. (D) Mean number of apoptotic cardiac myocytes per microscope field in the mouse myocardium. (E) Representative transthoracic echocardiograms of the mouse heart. (F) Mean EF value in the mouse myocardium. For each group: *n* = 5, three independent experiments. Statistical analysis was performed using one-way ANOVA. *p < 0.001 VS. control and sham; *p < 0.001 vs. AMI.

The effect of A_2B_AR blockade on histopathological changes in the mouse heart after LADCA ligation was examined by HE staining. The results showed that 24 h after LADCA ligation, the infarcted cardiac muscle exhibited a large number of inflammatory granulocytes infiltration, fibrous structure disorder, myocardial fibre swelling and rupture (Figure 3(A,B), Supplementary Figure 3). While A_2B_AR blockade alleviated myocardial injury after LADCA ligation. Similar results were found in TTC staining which showed that A_2B_AR blockade can significantly alleviate the area of myocardial infarction in AMI mice (Supplementary Figure 3).

Further TUNEL staining showed that A_2B_AR blockade significantly reduced cardiac myocyte apoptosis after AMI (Figure 3(C,D)). Moreover, we found that mice with AMI exhibited decreased cardiac systolic function (Figure 3(E,F), Supplementary Table 2) and that A_2B_AR blockade improved cardiac systolic function after MI.

These results suggested that A_2B_AR blockade effectively alleviates myocardial injury and improves cardiac systolic function after AMI.

### 3.4. Spleen-derived MDSC mobilisation after AMI

**Figure 4.**
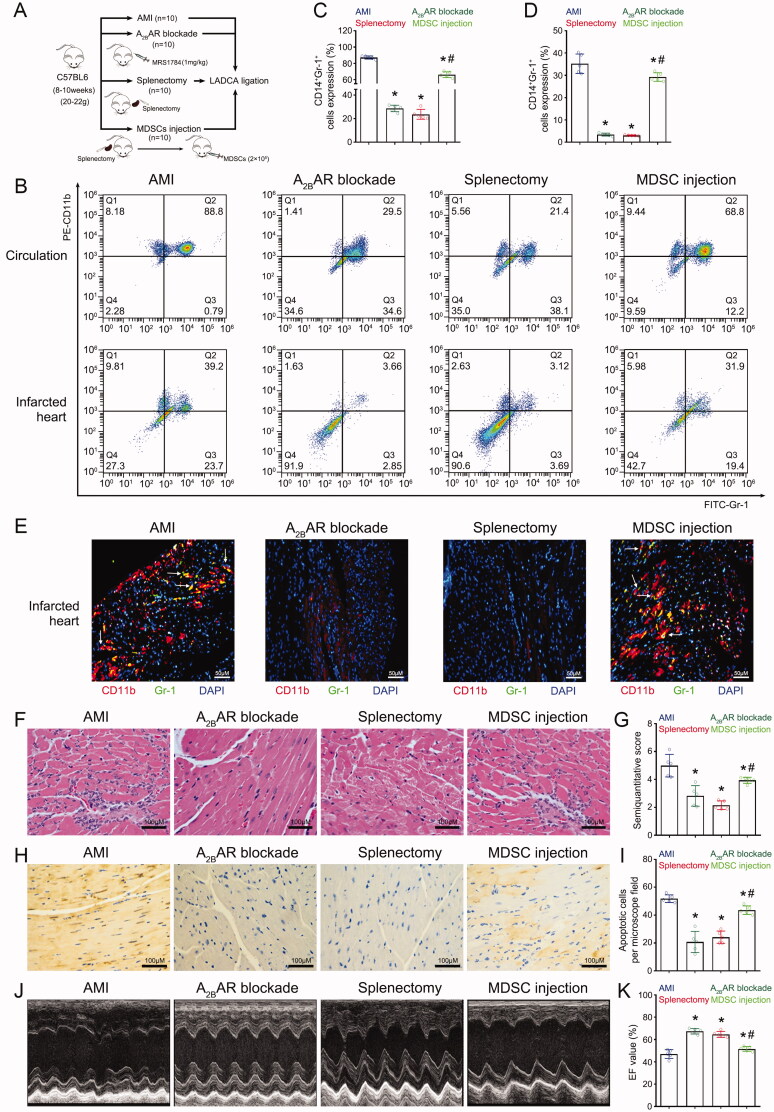
Spleen-derived MDSC mobilisation led to myocardial injury after AMI. (A) The different groups of mice. Male C57BL6 mice (age: 8–10 weeks, weight: 20–22 g) were randomly divided into 4 groups. The AMI group (*n* = 10) underwent LADCA ligation. The A_2B_AR blockade group (*n* = 10) received intraperitoneal injection of the A_2B_AR antagonist MRS1784 (1 mg/kg) before LADCA ligation. The splenectomy group (*n* = 10) underwent splenectomy before LADCA ligation. The MDSC injection group (*n* = 10) received isolated MDSC (2 × 10^5^) injection before LADCA ligation. All mice were housed in an SPF animal facility with unrestricted access to food and water. Samples, including blood and heart and spleen tissues, were harvested 24 h after the operation. (B) Representative flow cytometry images of MDSCs in the circulation and infarcted heart among the 4 groups. (C) Mean fluorescence intensity of CD11b^+^ CD14^–^ cells in the circulation of mice. (D) Mean fluorescence intensity of CD11b^+^ CD14^–^ cells in the infarcted mouse heart. (E) Representative immunofluorescence staining images of CD11b (red), Gr-1 (green), and DAPI (blue) staining in the infarcted mouse heart and merged images (right column). Both A_2B_AR blockade and splenectomy decreased the MDSC ratio, and MDSC injection increased the ratio of MDSCs in the infarcted hearts of mice with AMI. (F) Representative HE staining images of the infarcted mouse heart. (G) Mean semiquantitative scores of inflammatory cells in the mouse myocardium. (H) Representative images of myocardial apoptosis in the infarcted mouse heart. (I) Mean number of apoptotic cardiomyocytes per microscope field in the mouse myocardium. (J) Representative transthoracic echocardiograms of the mouse heart. (K) Mean EF of the mouse myocardium. For each group: *n* = 5, three independent experiments. For each group: *n* = 5 (A–D, F–K), *n* = 3 (E); three independent experiments. Statistical analysis was performed using one-way ANOVA. *p < 0.001 vs. the AMI group; *p < 0.001 vs. the A_2B_AR blockade and MDSC injection groups.

To explore whether the mobilised MDSCs in mice with AMI are derived from the spleen, splenectomy was performed before LADCA ligation (Figure 4(A)). We found that similar to A_2B_AR blockade, splenectomy significantly decreased the MDSC ratio in the circulation (Figure 4(B,C)). The MDSC ratio in the infarcted heart was significantly decreased after splenectomy, similar to after A_2B_AR blockade (Figure 4(B,C)).

To further verify that spleen-derived MDSC mobilisation led to myocardial injury after AMI, spleen-derived MDSCs (2 × 10^5^/ml) were isolated and injected into mice that underwent splenectomy via the medial canthus vein before LADCA ligation (Figure 4(A)). We found that compared to A_2B_AR blockade and splenectomy, MDSC injection significantly increased the MDSC ratio in the circulation (Figure 4(B,D)). The MDSC ratio in the infarcted heart was significantly increased after MDSC injection compared to after A_2B_AR blockade and splenectomy (Figure 4(B,D)).

Immunofluorescence also revealed that both A_2B_AR blockade and splenectomy decreased the MDSC ratio and that MDSC injection increased the ratio of MDSCs in the infarcted hearts of mice with AMI (Figure 4(E), Supplementary Figure 1). These results suggested that the spleen is the source of mobilised MDSCs in mice with AMI.

### 3.5. Spleen-derived MDSC mobilisation led to myocardial injury after AMI

We examined whether splenectomy affects myocardial injury and function in mice with AMI. We found that similar to A_2B_AR blockade, splenectomy alleviated myocardial injury (Figure 4(F,G), Supplementary Figure 3) and decreased the number of apoptotic cells (Figure 4(H,I)) in the infarcted hearts of mice with AMI. Cardiac ultrasound also showed that splenectomy had the same effect in preserving cardiac systolic function after AMI (Figure 4(J,K)).

Further study showed that compared to A_2B_AR blockade and splenectomy, MDSC injection aggravated myocardial injury (Figure 4(F,G), Supplementary Figure 3), increased the number of apoptotic cells (Figure 4(H,I)), and decreased cardiac systolic function (Figure 4(J,K)). These results suggested that spleen-derived MDSC mobilisation is the main cause of myocardial injury after AMI.

## 4. Discussion

In the present study, we revealed that spleen-derived MDSCs mobilise into the circulation and infarcted heart tissue, leading to myocardial cell apoptosis and tissue inflammatory damage and ultimately impairing cardiac systolic function. A_2B_AR blockade can inhibit myocardial cell apoptosis, alleviate myocardial inflammatory injury, and improve myocardial systolic function, mainly *via* prevention of spleen-derived MDSC mobilisation after AMI.

AMI is an acute fatal disease characterised by massive immune cell infiltration into the myocardium following ischaemia and reperfusion [35]. Exploring the characteristics of the immune cells infiltrating the myocardium and identifying relevant interventions can help prevent irreversible myocardial injury after MI. In this study, we first revealed that blocking A_2B_AR prevented spleen-derived MDSC mobilisation, alleviated myocardial injury, and improved the cardiac systolic function of mice subjected to LADCA ligation. This suggests that preventing the mobilisation of spleen-derived MDSCs is a potential strategy for AMI treatment.

MDSCs, including immature granulocytes, macrophages, and dendritic cells at different stages of differentiation, are well-known immunosuppressive cells that play a decisive role in many disease states [7]. A widely accepted viewpoint is that infiltrating MDSCs can secrete high levels of inflammatory cytokines to mediate uncontrolled inflammation and accelerate inflammatory damage to tissues and organs [36,37]. Recent studies have reported that MDSCs are mobilised and recruited to the infarcted myocardium in the acute stage of AMI, acting as primary infiltrating myeloid cells and contributing directly to the oxidative burst, secretion of proteolytic enzymes, and promotion of cardiomyocyte death [9,16,17]. Consistent with these previous studies, our study observed that the content of MDSCs was significantly increased in the circulation of AMI patients. This phenomenon was also observed in the mice subjected to LADCA ligation. Furthermore, our study also revealed that enhanced infiltration of MDSCs in the infarcted heart was associated with myocardial cell apoptosis and tissue inflammatory damage in mice subjected to LADCA ligation. In addition, we observed that the content of MDSCs was significantly decreased in the spleens of mice subjected to LADCA ligation. This result indicates that the spleen is a potential source of MDSCs during AMI.

The spleen is an easily accessible peripheral immune organ and is thought to be the source of large quantities of immunocytes, including MDSCs, lymphocytes and macrophages [38,39]. Various studies have indicated that spleen-derived CD11b^+^ Gr-1^+^ cells are the major contributors to inflammatory reactions and organ damage [15,16,40]. In this study, we found that splenectomy significantly decreased the MDSC ratio in the circulation and infarcted myocardium. These results further confirm that the spleen is the source of MDSCs during AMI. Interestingly, myocardial damage was mild in mice subjected to splenectomy before AMI. Considering that there are numerous immune-related cells in the spleen, spleen-derived MDSCs were isolated and injected intravenously into mice before LADCA ligation. The results showed that intravenous administration of MDSCs aggravated myocardial injury in mice subjected to LADCA ligation by inducing infiltration of MDSCs into the infarcted myocardium. All of these results demonstrate that spleen-derived MDSC mobilisation is an important cause of myocardial damage during AMI. Therefore, investigating the mechanism of spleen-derived MDSC mobilisation during AMI may provide a scientific basis for AMI treatment.

A_2B_AR is a G-protein coupled adenosine receptor that is widely expressed in haematopoietic cells and plays an important role in many pathophysiologic conditions [41–43]. A_2B_AR is expressed in Gr1^+^ cells, and evidence has revealed that A_2B_AR promotes the accumulation of intratumoral CD11b^+^ Gr1^+^ cells in many tumour models and that pharmacological blockade of A_2B_AR significantly reduces tumour growth [25,29

]. Some biomolecules and synthetic drugs protect organs, including the heart, liver, and brain, against ischaemic–reperfusion injury *via* A_2B_AR signalling [44–47].

In this study, we found that A_2B_AR mRNA was highly expressed in the mononuclear cells of AMI patients. This result indicates that A_2B_AR may be involved in MDSC mobilisation during AMI. To further explore this issue, MRS-1754, a selective A_2B_AR antagonist [48,49], was intraperitoneally injected into mice before LADCA ligation. We found that blocking A_2B_AR with MRS-1754 prevented spleen-derived MDSC mobilisation and improved the cardiac systolic function of mice with LADCA ligation.

## 5. Conclusion

In our study, we first revealed that A_2B_AR plays a pivotal role in myocardial injury by facilitating spleen-derived MDSC mobilisation during the acute phase of AMI. Blocking A_2B_AR with MRS-1754 improves cardiac systolic function. This effect is, at least in part, dependent on impaired spleen-derived MDSC mobilisation. Therefore, our study suggested that A_2B_AR-mediated spleen-derived MDSC mobilisation is a potential therapeutic target for AMI.

## 6. Limitations

There are two major limitations to this study. First, we did not explore the role of MDSC subtypes in AMI. Changes in the A_2B_AR signalling pathway in MDSCs need to be further studied. Furthermore, we only explored the mechanism of MDSC mobilisation within 24 h after AMI. Third, small number of patients were included in this study. It may affect the credibility of the results. Therefore, we would conduct a larger sample size study to verify the results. Therefore, the mechanism of MDSC mobilisation during the recovery period after AMI needs to be further explored.

## Supplementary Material

Supplemental Material

## Data Availability

The datasets used or analysed during the current study are available from the corresponding author on reasonable request.
