# Supplementary material for: Blocking the A2B adenosine receptor alleviates myocardial damage by inhibiting spleen-derived MDSC mobilisation after acute myocardial infarction
Source: Ann Med. 2022 Jun 8;54(1):1616–26. doi: 10.1080/07853890.2022.2084153 (PMC9186371; doi:10.1080/07853890.2022.2084153)
Supplement: Supplemental Material [file IANN_A_2084153_SM2883.zip › Supplemental files/Supplementary Figures.docx]

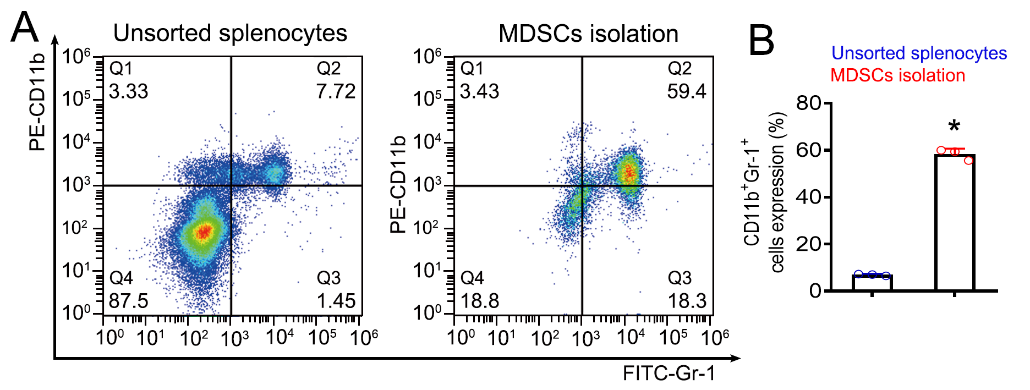


**Supplementary Figure 1. FACS was used to evaluate the separation efficiency of MDSCs isolated from the spleen.** The separation efficiency of isolated MDSCs was approximately 59%. For each group: n = 3, three independent experiments. Statistical analysis was performed using Student’s t test. ^*^*P*< 0.001 vs. unsorted splenocytes.


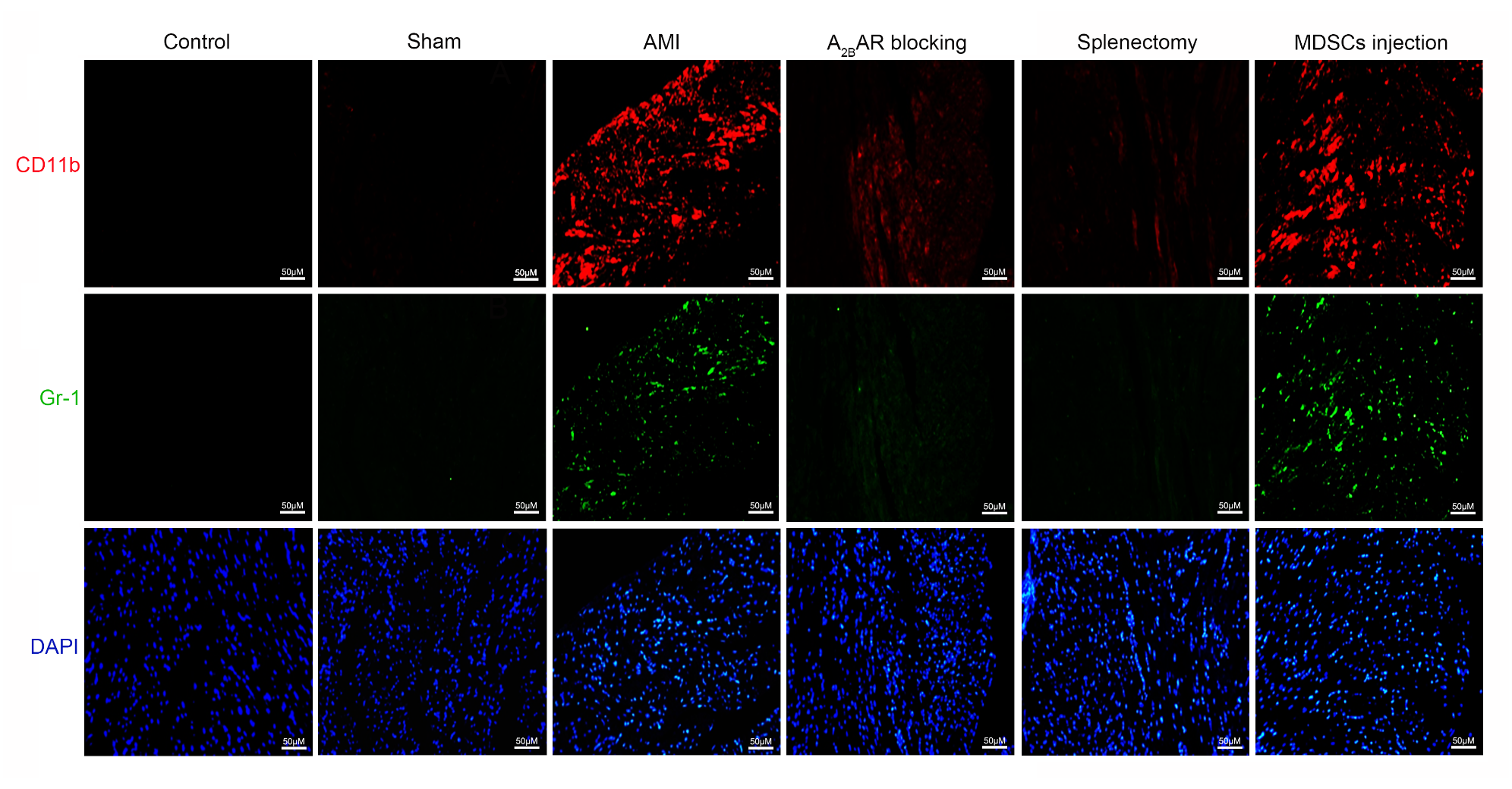


**Supplementary Figure 2. Representative immunofluorescence images of MDSCs in the infarcted mouse heart.** The percentage of MDSCs was increased in the infarcted mouse heart. Both A_2B_AR blockade and splenectomy decreased the MDSC ratio, and MDSC injection increased the ratio of MDSCs in the infarcted hearts of mice with AMI. Representative images of CD11b (red), Gr-1 (green), and DAPI (blue) staining in the infarcted mouse heart. For each group: n = 3.


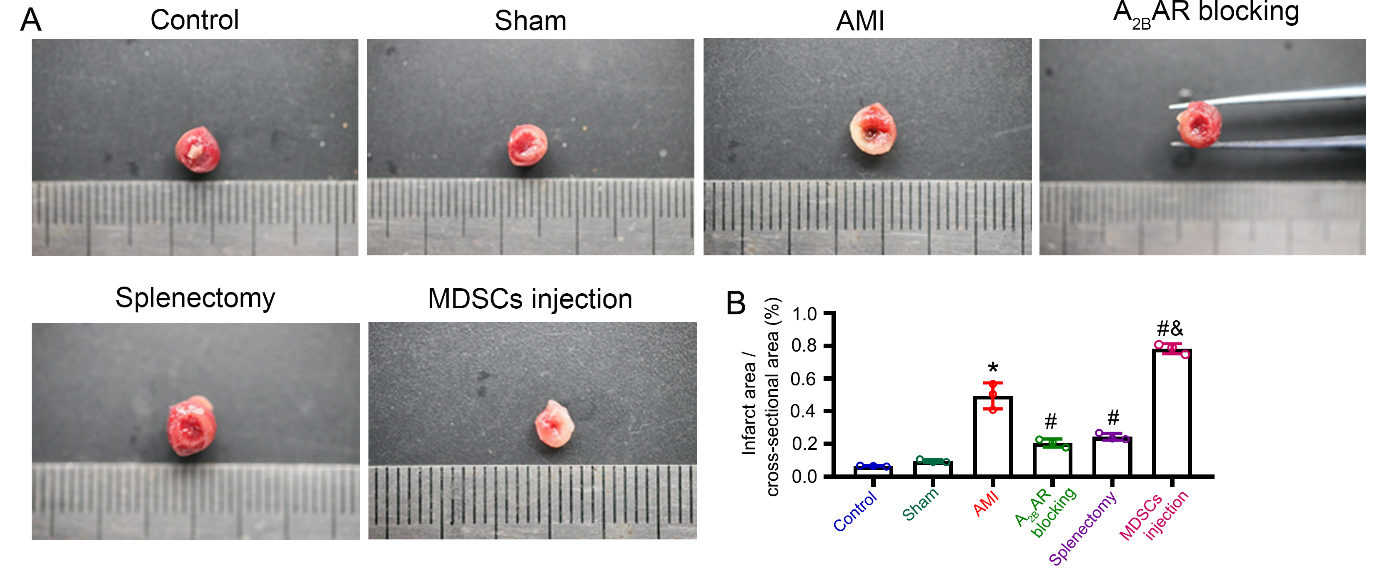


**Supplementary Figure 3. Representative images of the area of myocardial infarction that detected by TTC stanning. A** Representative TTC staining images of the infarcted mouse heart. **B** Mean ratio of the infarct area to the corresponding cardiac cross-sectional area in the mouse myocardium. For each group: n = 3, three independent experiments. Statistical analysis was performed using one-way ANOVA. ^*^*P*< 0.001 vs. unsorted splenocytes.
