# Supplementary material for: Blocking the A2B adenosine receptor alleviates myocardial damage by inhibiting spleen-derived MDSC mobilisation after acute myocardial infarction
Source: Ann Med. 2022 Jun 8;54(1):1616–26. doi: 10.1080/07853890.2022.2084153 (PMC9186371; doi:10.1080/07853890.2022.2084153)
Supplement: Supplemental Material [file IANN_A_2084153_SM2883.zip › Supplemental files/Supplementary Tables.docx]

**Supplementary Table 1. Basic data of included patients.**

|  | **Control**  **(n=10)** | **AMI**  **(n=10)** | ***P* value** |
| --- | --- | --- | --- |
| Female [n(%)] | 3 (30.0) | 4 (40.0) | 0.639 |
| Age (years) | 60.30±6.09 | 63.70±5.68 | 0.213 |
| Hypertension [n(%)] | 6 (60.0) | 7 (70.0) | 0.639 |
| Diabetes [n(%)] | 2 (20.0) | 3 (30.0) | 0.606 |

Note: AMI: acute myocardial infarction. Data were calculated by Student’s t test.

**Supplementary Table 2. Ultrasound data about myocardial systolic function**.

|  | **Control** | **Sham** | **AMI** | **A_2B_AR**  **blocking** | **Splenectomy** | **MDSC**  **injection** |
| --- | --- | --- | --- | --- | --- | --- |
| EF, % | 79.2±2.3 | 80.0±1.6 | 47.0±3.9^a^ | 67.4±2.4^b^ | 64.6±2.7^b^ | 51.6±2.1^c^ |
| LVFS, % | 42.2±2.0 | 41.0±2.3 | 27.0±2.0^a^ | 38.7±2.3^b^ | 39.2±2.2^b^ | 28.4±3.9^c^ |
| LVIDd,mm | 3.88±0.21 | 3.84±0.12 | 4.40±0.35^a^ | 4.02±0.16^b^ | 3.98±0.21^b^ | 4.24±0.15^c^ |
| LVIDs,mm | 2.17±0.19 | 2.13±0.12 | 2.98±0.17^a^ | 2.41±0.26^b^ | 2.43±0.13^b^ | 2.79±0.31^c^ |

Note: AMI: acute myocardial infarction; A_2B_AR: A_2B_ adenosine receptor; MDSC: Myeloid-derived suppressor cell; EF: ejection fraction; LVFS: left ventricular shortening; LVIDd: left ventricular end diastolic diameter; LVIDs: left ventricular systolic inner diameter. Data were calculated by one-way ANOVA. ^a^: P<0.05 vs Control and Sham; ^b^: P<0.05 vs AMI; ^c^: P>0.05 vs AMI.
